# Suffering caused by care—elderly patients’ experiences in community care

**DOI:** 10.3402/qhw.v8i0.20603

**Published:** 2013-11-20

**Authors:** Rune Svanström, Annelie Johansson Sundler, Mia Berglund, Lars Westin

**Affiliations:** 1 School of Life Sciences, University of Skövde, Skövde, Sweden; 2 School of Health, Care and Social Welfare, Mälardalen University, Eskilstuna, Sweden

**Keywords:** Elderly, care, dementia, lifeworld, patient experiences, suffering

## Abstract

**Background:**

Growing old involves many changes in life and implies an increased risks of illness and different forms of disabilities. Life may change in a radical way when a person gets a disease like dementia or moves to a nursing home due to disabilities or needs. In both cases, it often leads to an increased dependency on care where the patient becomes exposed and vulnerable and thereby at a higher risk for experiencing different forms of suffering.

**Aim:**

The aim of this study was to elucidate and gain a deeper understanding of elderly patients’ experiences of suffering in relation to community care in nursing homes and home care services.

**Materials and methods:**

A lifeworld hermeneutical approach was used. Phenomenological interviews and conversations with an open approach were conducted and analysed with a focus on meanings.

**Findings:**

The findings were presented in four main themes; an absence of the other in care, an absence of dialogues, a sense of alienation and a sense of insecurity. The findings in this study revealed that persons who were cared for in nursing homes and home care services sometimes were exposed to an unnecessary suffering. The suffering sometimes was caused by various caring actions, that is, unnecessary suffering. The suffering caused by care that aroused was due to caregiver’s inability to be present, to show their face, and truly meet the patient.

**Conclusion:**

Suffering from care increased the elderly patients’ feelings of insecurity, loneliness, and alienation; this seemed to be the foundation for patients’ experiences of being outside a human community. There was a lack of knowledge and understanding about the patient’s lifeworld.

This study, based on patients’ and spouses’ stories of encounters with caregivers in community care contexts, intends to illuminate the meaning of suffering caused by this care. Patient suffering has been described as a basic motive for caring (Eriksson, 1997, 2002). Erikson’s theoretical model describes the suffering in three forms: as related to illness, as related to care, and as related to life. The primary objective of caring is to recognize and alleviate patient suffering, even if the care, to achieve health, sometimes may create a temporary, but necessary, suffering caused by care (Dahlberg & Segesten, 2010), for example when a psychotic person is forced into psychiatric care.

From a caring science perspective, illness or disease, as well as health, need to be understood as an embodied experience. Health and illness - subjective experiences that cannot be reduced merely to the physical attributes of a disease - involve both the body and the lifeworld of the patient. Philosopher Merleau-Ponty (2002/1945) emphasizes human existence in terms of “the subjective body.” The lived body is our “anchorage” in the world; not only is it something concrete and biological, but also it is through this bodily being that we get access to the world and our everyday life. Our bodily being is also a social being, and in our bodily encounter with other human beings, Lévinas (2005/1961) points out the face as the key to discovering what a human being is. When we meet the other human being and see the face of the other, we can learn about our self as human beings but also who the other is (p. 39). This relational perspective seems important in caregiving when it comes to the patient’s subjective experience of suffering, health, and well-being.

According to Eriksson (1994), suffering caused by care occurs when the patient’s dignity and human values have been disregarded. Being in need of care will most often be associated with a certain degree of suffering (Fagerström, Eriksson, & Bergbom-Engberg, 1998). The patient’s subjective experience raises important questions in relation to well-being and suffering. It has been argued that recognizing the patient’s own perspective is valuable and that this is crucial for actively including the patient in care and, thereby, reducing the risk of having negative experiences from care in different care situations (Dahlberg & Segesten, 2010). Inadequate communication between caregiver and patient can create suffering caused by care in the form of a sense of insecurity for the patient.

Growing old involves many changes in life and implies increased risks of illness and different forms of disabilities. Life may change in a radical way when a person gets a disease such as dementia or moves to a nursing home due to disabilities or needs. In both cases, it often leads to an increased dependency on care (Nordberg et al., 2007), where the patient becomes exposed and vulnerable and thereby at a higher risk for experiencing different forms of suffering (Dahlberg & Segesten, 2010).

Dementia often results in different forms of suffering (Nygård & Borell, 1998; Nygård & Johansson, 2001; Nygård & Starkhammar, 2003; Phinney & Chesla, 2003; Svanström & Dahlberg, 2004; Svanström & Sundler, 2013). The disease affects the person, especially in moderate and severe stages of dementia (SBU, 2008), and it becomes more and more problematic for the individual to anchor him or herself in the world, which leads to difficulties in carrying out everyday life routines (Nygård & Borell, 1998; Phinney & Chesla, 2003; Svanström & Dahlberg, 2004; Svanström & Sundler, 2013). The person with dementia becomes dependent on care daily, which in many cases creates problems that cause the person to move to a nursing home. The need for care also affects relatives, who often invest great efforts in taking responsibility for the care of the ill individual (Almberg, Grafström, & Winblad, 1997; Hellström, Nolan, & Lundh, 2007; Jansson, Nordberg, & Grafström, 2001; O’Shaughnessy, Lee, & Lintern, 2010; Söderlund, 2004; Svanström & Dahlberg, 2004).

Moving into a nursing home and becoming a resident is a significant change that can influence both the residents and their relatives. It is a challenge for all care workers to promote well-being and alleviate suffering in the context of the person’s new life situation, which often is affected by complex care needs. To alleviate the person suffering from these changes, care should include physical, social, and existential dimensions (Bergland & Kirkevold, 2001). One important aspect in meeting the person’s needs is good communication in the encounter between nurses, nursing home residents, and their relatives (Andersson, Pettersson, & Sidenwall, 2007; Berglund, 2007; Westin & Danielson, 2007). A study by Gijsberts, van Der Steen, Muller, Hertogh, and Deliense (2013) showed that there was a lack of communication about spiritual care in Dutch nursing homes. Spiritual issues were addressed only informally and were not a part of the formal care process. Oosterveld-Vlug et al. (2013) showed in a study about residents’ experiences of personal dignity that many residents felt discarded and not taken seriously, simply because of their age or illness. Waiting for help, being dictated by nurses, and not receiving enough attention could undermine personal dignity. Therefore, it is important that nurses pay attention to residents’ needs of intersubjectivity and to interact in a social context. It is also important for nurses to reflect on their part and responsibility in encounters with nursing home residents (Wadensten, 2007; Westin & Danielson, 2007). Other important aspects for the residents are safety and confirmation as being someone in the nursing home and having meaningful activities in their everyday life (Cook, 2006; Hjaltadóttir & Gustafsdóttir, 2007; Westin & Danielson, 2007).

Having dementia in moderate or severe stages or moving into a nursing home can most likely affect the person’s existence at a deeper level. It can be assumed that knowledge about the existential dimensions of illness and well-being is essential in care to avoid unnecessary suffering. To develop strategies that can alleviate suffering, it is important to investigate those existential dimensions of illness and well-being from the individual’s perspective. In human existence, suffering is related to life itself or to different diseases that at times cannot be avoided, but when it comes to suffering related to care, the question of how this kind of suffering can be avoided remains. From this point of view, it appears important to describe how patients experience the phenomenon of suffering related to care. The aim of this study was to elucidate and gain a deeper understanding of elderly patients’ experiences of suffering in relation to community care in nursing homes and home-care services.

## Materials and methods

In this study, we used a lifeworld hermeneutical approach (Dahlberg, Dahlberg, & Nyström, 2008/2001) that supports investigations about phenomena in our everyday lives. When people who are suffering from long-term diseases and simultaneously dependent on care are interviewed, there is an opportunity to capture their everyday life experiences (i.e., parts of their lifeworld) (Giorgi, 2009). In interviews conducted in earlier studies, we saw a suffering that suggested that caring sometimes could cause suffering. To further illuminate this suffering, this study was conducted as part of a project on suffering from care. A secondary analysis was performed, which may allow researchers to apply a new research question to data already collected (Heaton, 2004). The reuse of data originally collected in earlier studies was critically discussed by the researcher, who found it unethical not to further explore the suffering that called for our attention.

The aforementioned studies were approved by the regional ethical committee in Gothenburg, Sweden (L 263-98; Ö 403-01; Ö 446-03), and both this study and the former studies have followed the Declaration of Helsinki.

### Participants and data material

Interviews with participants recruited from primary and community care were analysed in this study. The participants had been guaranteed confidentiality and were informed that participation was voluntary. All participants had given their informed consent.

The participants were between 72 and 90 years of age, and they had lived with a long-term disease such as post-stroke impairments or dementia for several years or as the spouse of a person with dementia for several years. The participants, except for the spouses, had a manifest healthcare need; some were living in nursing homes, and some were receiving home-care services from caregivers to manage daily life.

The data material consisted of qualitative interviews and conversations with 25 participants. Data had been gathered through interviews with patients living in nursing homes and with patients with dementia having a manifest care need and their spouses living together at home. Data had also been gathered through conversations with patients with dementia having a manifest care need and living at home alone. All interviews and conversations contained detailed and different experiences of when they suffered from care, descriptions which then were used in this analysis. In these interviews and conversations, the focus had been on the participants’ expressions and narratives of lived experiences from their everyday life. Experiences described from people with different diseases, as well as from shifting contexts, formed a richness of variation in the data material.

### Analysis

The analysis was inspired by Dahlberg et al. (2008/2001) and carried out in continuous dialogue with the text; in the analysis, the researchers moved back and forth in all interview texts. The analysis started with the researchers first reading all of the interviews to acquire a general sense of the patients’ experiences of suffering. In this initial phase, interview texts were read several times. The next step in the analysis was a search for patterns and nuances of qualitative meanings of suffering. After that, the text was condensed, and meanings found in the text were discussed and structured in themes. The meaning of the themes was described, and four patient stories were constructed to illustrate these meanings of suffering caused by care. The patient stories were formulated based on descriptions in the interviews. The rationale for creating patient stories were that the stories could illustrate the data and their meaning in new ways. Another reason was to facilitate a deeper understanding of the phenomenon being studied. Dahlberg et al. (2008/2001) stress the importance of being creative and open in the analysis in order not to define the findings too quickly.

### The four patient stories

In the four stories, the participants were given fictive names to protect their identity. The four stories were used to illustrate lived experiences, even if the meanings described in the themes occurred from more than these four persons’ interviews. The four patients illustrated in the stories consist of two men, one woman, and one couple. The men, Stig and Arnold, lived in a nursing home; the woman, Anna, lived alone in her apartment; and the couple, Astrid and George, lived in an apartment.

Stig needed daily care; after his leg amputation, an exuding wound developed on his stump, which was something that gave him feelings of disgust. Arnold did not need the same amount of care, but he was determined and knew what he wanted, something that eventually came into conflict with the caregiver’s view. Both men have had many contacts with caregivers every day and even at nights. Anna, who had been diagnosed with dementia, was visited by caregivers several times a day and visited a day-care centre several times a week. She received assistance with hygiene, meals, laundry, and cleaning in the home. George, also diagnosed with dementia, had no ongoing care contacts, as he felt no need for them. Instead, it was his wife, Astrid, who was responsible for his care, a care which primarily was about being near George and answering his repeated questions. The term “caregiver” is used as a collective name for level-two nurses, auxiliary nurses, registered nurses, and social workers in this study.

## Findings

The findings reveal that persons who were cared for in nursing homes and home-care services sometimes were exposed to unnecessary suffering. The suffering could be caused by various caring actions, that is, unnecessary suffering. The suffering caused by care that arose seemed to be related to caregivers’ inability to be present, to show their face, and to truly encounter the patient. Suffering from care increased the elderly patients’ feelings of insecurity, loneliness, and alienation; this seemed to be the foundation for patients’ experiences of being outside of a human community. There was a lack of knowledge and understanding about the patients’ lifeworld. The findings are further described in this article in these four themes: an absence of the other in care, an absence of dialogues, a sense of alienation, and a sense of insecurity.

### An absence of the other in care

The encounter between the caregiver and the patient seems to bring an indication of care where the caregiver sometimes becomes absent for the patient. There was an uncertainty for the patients in the nursing home and sometimes a fear of future meetings in that the patients did not know what to expect in the next meeting. Fixed times for meetings were not plentiful, and the patient was provided with little knowledge about care schedules, yet someone came and helped with various activities of daily living (ADL) tasks. The patients did not know which caregiver would enter the room, which made them wonder if the caregiver would treat them as a person or simply as someone who needs care. That is, will it be a caregiver who is not open to human contact - a caregiver who only cares about the task that is pre-formulated and about to be performed?

Stig talked about how young female caregivers were more occupied with themselves and their own appearance rather than taking care of him. This raised profound existential questions about himself, and he did get a feeling of disgust as he lied with the exuding wound on his stump. He thought that this was the reason why caregivers did not see him and did not want to make contact or initiate dialogue with him. As he put it, “Yes, you don’t really exist; you become more like a ghost.”

For the person with dementia who lived alone, the situation was different. The patient did not know when the caregiver, that is, the other one, would arrive, or even if someone would come to visit. The sense of not knowing seemed to make the patient passive; there was nothing to wait for or prepare for. This was evident in Anna’s story. She did get a strong sense of loneliness when she did not know if anyone was coming. In the conversation with the interviewer, it became obvious that home-care services did not intervene and leave a lasting impression in her lifeworld.

Anna: I am alone. (Interviewer: But when you’re in this loneliness, don’t you think that someone will come to me in a few hours, someone I can talk to for a while, and tomorrow I will go to the day care centre, (No) and meet people there?) No, I … live in the present. Unfortunately perhaps, but I do. (Interviewer: How far does your present moment reach?) My present moment is today, and what happened yesterday maybe, I won’t remember everything. No, you know, my memory is gone.

When the caregiver only carried out the task without the needs of the person with dementia in focus, there was an obvious risk that the care did not leave any sustainable traces in the patient’s world, and a feeling of loneliness became obvious. This feeling tended to contribute to a passive life. For the person with dementia who lives alone, the possibility of good meetings often seemed to occur at the day care centre, where the patient was in a context that gave meaning. This context also was at hand in the person’s home but did not seem to be used by the caregivers.

The couple living at home strived on without any meetings or help from the home-care service. The situation was worsening slowly, and Astrid felt like she no longer could leave her husband, not even for a short while, as, for example, to go to the hairdresser. In this situation, no one from the home-care service had visited the couple or tried to find out about the couple’s situation. No one but the couple’s daughter realized how tired Astrid was, a circumstance that made Astrid call and ask for relief. Even after their difficult situation was discovered, nobody from the home-care service went to their home for a visit. The couple did stand alone, with no one investigating if there was any continued need for care. Astrid did not know her rights when she was talking to the interviewer, and he asked what she thought about the meeting with the home-care service.

Astrid: I have only good things to say. I have nothing to complain about. They will help us … sigh … During Whitsun week, I needed to go and perm my hair and I called and asked if they could come and be here with him while I was away. But then, I got him into short-term care and then I could do it. The home care service has never been here, but they would have come. So, I cannot complain about them.

Her husband went to short-term care, but Astrid could not hand over the responsibility so she visited him. Since he wanted to come home, she brought him home again after only 2 days. It appears from Astrid’s statement that she found it difficult to make demands on the home-care services. It also appeared that there were no meetings to create a dialogue through which needs could be expressed. The data suggest that there was no ongoing relationship between the couple and the home-care services. This means that there were no clear structures in the care, and that it was up to the patient or a spouse to assert his or her rights. It seemed to be the discretion of the caregivers that determined the approach to and the content of care. This gives a picture of a passive home-care organization that did not really recognize basic human needs.

### An absence of dialogues

Arnold: Well, they do not listen to what I say and then I do not listen to them either, that is the way it is. Some people you may connect with every time you meet them and with others less so. Yes, and with some people you may never connect with. (Interviewer: But what does this connection mean to you, when you really feel connected, when the caregiver really listens to you Arnold?) Well, but this connection really means a lot to me. It is worth a great deal.

This conversation took place between Arnold and the interviewer when they talked about his experiences of encounters with the caregivers; it was experienced as difficult to make contact with the caregivers. He terms those who he felt were difficult to connect with as “unjust”. It seemed that the day itself decided the form of the meeting, including the present mood of the caregiver. Both Stig’s and Arnold’s stories suggest an absence of an ongoing dialogue. It was rather that the dialogue occurred in the moment, when the caregiver was open to a positive meeting; or, as Arnold described the situation, “They joke with me to make me happy. They listen to me and make jokes with me and then you feel as you really are a human being.”

All of George’s healthcare needs exhausted Astrid. She frequently struggled with responding to George’s repeated questions. Sometimes, she did get irritated and lost her patience with him, but repented shortly thereafter. She stood alone in her care work, and there was a great and acute risk of him ending up in an institution such as a nursing home. Meanwhile, Astrid expressed her situation as follows: “I’ll try as long as I can.”

The home-care service became aware of the couple’s difficult situation after Astrid’s cry for help. It is common knowledge that with dementia, situations such as this one rarely improve. Nonetheless, the home-care service seemed to neglect this problem, had acted as if there was no problem, and had not visited the couple. There seemed to be no sense of responsibility from the home-care service’s point of view to establish a dialogue with the couple. In this case, the responsibility seemed to fall entirely on the couple, and especially on Astrid.

For Anna, who lived alone, there seemed to be no satisfactory dialogue either. What characterized caring seemed to be the fast care meetings, where the caregivers always were on the run to the next patient or where the caregivers chose to focus on their own problems, rather than an encounter that could turn into a dialogue about the patient’s life situation. Often what was in focus was providing the patient with medicine or food, or quickly tidying the apartment. The patient would then be without the important human contact when common tasks were performed.

Performing tasks together in a dialogue can anchor the patient in a context, in the everyday world. Instead, loneliness became amplified in the contact with the caregiver. There were strong emotions that took over and dominated the patient’s life, something that was evident in Anna’s story. Caregivers served her food and then proceeded with their other tasks; as a result, Anna occasionally did not eat. In the conversation with the interviewer, she suddenly became aware of her own plight.

Anna: No, they are in such a hurry. (Interviewer: The intention is that they give you food (yes, yes) and that you should eat it. But, here it is like you just you get food placed in front of you, and you don’t eat it.) Well gee, yeah, as long as they are present here…. I eat, of course. Yes. But sitting there alone, you know, day in and day out, there is something terrible. I’ve never been alone all my life, as I say. And now. No, I don’t want not be a part of it anymore. Now it is enough. (Interviewer: What do you mean when you tell me that?) Yeah…. my life can end now. It is enough now.

The consequences of an absent dialogue become obvious: the patient’s basic care need does not become fulfilled. When caregivers are in a hurry, the patient’s sublime voice may become only a whisper that no one listens to.

This shows the value of the caregiver in seeing and acknowledging the patient; this creates the conditions for a dialogue. Stig, who lived in a nursing home, said the following.

Stig: Yes it is, yes it is the connection, the connection through the eyes. It is in the manner they offer themselves, they are sympathetic above all, they want to try to understand me, that says it all really.

Human dialogue includes both speech and intersubjectivity where both interlocutors see the other’s face. When a person’s face was visible, it showed his or her interlocutor who he or she is, and in the other one’s face, the person can see who his or her interlocutor is. This contributes to a type of confirmation and thus a sense of human identity. Both a person with dementia with a manifest care need who lives alone and a couple where one person has dementia with a manifest care need have difficulties with their identity. This also applies to people who have moved to nursing homes. When they move from their home, they are outside of their usual context. There will be new procedures and new environments, which can create feelings of insecurity, loneliness, and alienation. Because of this, the occurrence of intersubjectivity between caregivers and patients was of great importance for these patients.

### A sense of alienation

Being an active member of society becomes difficult not only for persons with dementia with a manifest care need but also for their partners. Sustaining life itself may become more than enough. For those who lived in nursing homes, there were also limited possibilities for an active life in society. Disability limited the possibility to be in one’s known contexts, and the caring approach may also contribute to a sense of alienation. The feeling of being outside of a human communion became strong when the care in a nursing home seemed to be, above all, about tasks to be completed. In Stig’s story, this was obvious when he shared examples of how caregivers came to his room to perform some tasks without addressing him, consequently not acknowledging him as a person. Stig would like to talk to his caregivers, but this did not occur often. The feeling of being alone became powerful even though he met caregivers frequently. In his words, “Well, you feel lonely, very lonely, well I say, you really do.”

The person with dementia with a manifest care need who lives alone was in a similar situation. The visits from the home-care services were often short and dealt with different tasks. The caregiver may be in a hurry and will not invite the patient to participate in the tasks. Caregivers said that they had much to do, and it was difficult to make any demands on them about the content of the visits. Here, feelings of loneliness became as obvious for the patient living at home as for the patient in a nursing home. Anna talked about the brief meetings with the caregivers and that she did not feel involved in what happened in the home. The caregiver was a welcome companion, but the lack of conversation about the patient’s lifeworld created strong feelings of loneliness and alienation. When Anna talked about her life and youth, her feelings of loneliness were reduced, but when the pace of the caregivers was fast and expressed a heavy workload, this often did not leave any room for her stories.

Anna: But they are in such a hurry when they come. They will never sit down like this and talk to me; instead, they are running in and out and … and vacuuming and cleaning and.… They are not real company and I like, I like to converse. You know? (Interviewer: Yes, yes, I know that. [Anna laughs]. But what is it like for you, when they do not stay?) Well yes, I know, but they have so much to do, they cannot stay and converse with me. I know this; they have so much to do, so I do not demand it … but I miss it.

The couple also has had a hard time; they easily became isolated in their home, making them feel alienated. The planning of the care for the couple was non-existent, and the home-care services were not present; no one from the home-care service had been able to help the couple. They did not get any support and were left in solitude, and when the care was absent, their loneliness and vulnerability were obvious, despite them being two persons who were together.

(Interviewer: What’s most negative about the disease?) Astrid: sigh … Well yes, it is that you become lonely. (Interviewer: What do you mean, lonely?) Yes, for example, I have to give up attending my needlecraft club meetings, and … now for example when bingo starts, if he doesn’t want to go, then I have to stay home. That’s the way it is.

### A sense of insecurity

The stories reveal insecurity among the patients. Stig, for example, had no opportunity to choose the outcomes of his encounters. One day, there was a caregiver who sees and confirms him, and the next time there was another who did not give him any confirmation at all; he became a nobody. Arnold told similar stories: At one time when he had to go to the bathroom in the night, he was denied this. By being stubborn and getting angry, he did get help in the end.

Arnold: Then I thought: ‘Now you have to speak up’. (Interviewer: How did it go?) Well it worked out in a good way in the end, but it’s hard not being listened to. (Interviewer: Yes, but what did you do to get help?) I told them off, and then I got help, but it was sad that they did not listen to me at once.

In Anna’s case, there were similar experiences of an unsecure everyday life. She was not given support in performing the everyday chores and in actively using her home. Her life became passive with feelings of loneliness and insecurity. One way to escape from all of this was by going to bed and sleeping. This inactive life affected her health negatively. In the case of George and Astrid, there was a similar problem. Their existence was fragile. If something happened to Astrid’s health, this would mean that George would not cope at home on his own. This created an unsafe situation for both Astrid and George. He would probably end up in an institution very quickly if Astrid’s care should be discontinued. The dialogue with the home-care services did not exist, and any caregiving that would support the couple and create basic security in their everyday life was absent. This left the spouse with overwhelming feelings of grief over a responsibility that she knew she could not keep.

(Interviewer: What do you know about the future with this disease and life with this disease?) Astrid: Well, the only thing I know is that I will go on like this as long as I can manage. Then, and then there is nothing to choose about: he has to move to a care institution. (Interviewer: You mean that he has to move somewhere else then?) Well, first of all, he may have to move into the short-term care. So they have told me, I only have to call them. But, as long as I can manage I will try. (Interviewer: For how long will you manage?) Well, I do not know. (Interviewer: What are your feelings on a day like this?) sigh … Well, today I feel pretty good, but sometimes it’s hopeless. (Interviewer: For how long have you had these feeling of hopelessness?) sigh, Well, maybe for about six months. (Interviewer: What is it like for you to live with this kind of despair, for such a long time?) Well, there is no quality of life, it’s not … [Astrid shortly thereafter burst into tears].

## Discussion

The findings in this study reveal that persons who are cared for in a nursing home and persons with dementia with a manifest care need being cared for at home sometimes were exposed to an unnecessary suffering, a suffering caused by care. This increased the patients’ experiences of insecurity, loneliness, and alienation, and this can be understood as experiences of feelings of standing outside of a human community. The basis of the experiences was encounters without someone to talk to. The patients in this study experienced not being secure about when, or if, the caregiver would open an avenue for communion. In fact, no one can know truly when this could happen, but for these actual patients, the unclear and insecure situation became more serious because of their vulnerability and their dependence on help from caregivers. Suffering from care like for these patients has been described elsewhere, as, for example, in hospital contexts (Berglund, Westin, Svanström, & Johansson Sundler, 2012). Unnecessarily suffering from care seems to appear in different caring situations, and not solely in nursing homes or in home-care services.

If there is a culture of care that does not allow the caregiver to confirm the patient in every single care action, there may be a lack of a holistic view on the care and at the same time a lack of respect for human dignity. Kasén, Nordman, Lindholm, and Eriksson (2008) argued that a rigid care organization with an unreflective attitude of the caregiver in his or her encounters with the patient can create suffering caused by care. The basis for this lack of caring was the caregiver’s view of humanity in which the patient sometimes became an object. To be seen as an object, without being involved in a dialogue, may create a feeling of being alone and isolated in which the patient does not get invited into a human community. The findings in this study showed that everyday life became insecure for the participants, disempowering them, which made their everyday life become passive. Life was occupied with feelings of loneliness, a lack of identity, and a sense of alienation, created by the absence of confirmatory care. This weakened the patients’ and even their partners’ identities and raised questions about if and how the care can change the culture into a facilitating organization that enables the patient to feel secure and truly at home in their home. In this study, patients experienced a sense of alienation when feeling lonely and not connected with or confirmed by the caregiver. When the other’s face is absent in the caring encounter, the patient may not feel confirmed as a human being. This can give a non-existent, intersubjective dialogue. Similar to the findings in this study, Dahlberg (2007) describes involuntary loneliness as a feeling of standing alone and outside of connectedness to others. When connecting with others, this loneliness can disappear.

A way to confirm others is by showing the face when meeting other persons (e.g., to be truly present). This was highlighted by Lévinas (2005/1961), who meant that the face gives us the recognition of the other, whose otherness then will appear. The recognition of the other’s otherness will appear in the moment. This was not instant and needs constant replenishment to be kept alive, or as Lévinas (2005/1961) put it, “The I is not a being that always remains the same, but is the being whose existing consists in identifying itself, in recovering its identity throughout all that happens to it” (p. 36). To have an identity means to be at home with oneself and to be an “I can.”

The patient’s own identity is an important part of caring for patients with dementia. To have an identity is, according to Kitwood (1997), to know who one is; it involves maintaining a sense of continuity with the past and some kind of consistency across the course of one’s present life. Attachment is another aspect of importance in care. The loss of primary attachment can undermine one’s sense of security. These aspects must therefore be suggested as important in the care of people suffering from dementia and probably also in the care of patients in nursing homes. Brown and Shlosberg (2006) have concluded that the occurrence of attachment behaviours among people with dementia who live at home with a caregiver is not well understood, which is an important area for further research. The patients in this study had difficulties with presenting their needs, especially when it came to expressing their desires. Their sublime wish, about the possibility to encounter the caregiver to feel confirmed and know that you are an accounted human being, was an unfulfilled desire. This left a broken identification process where the person seemed to have difficulties in grasping his or her identity and personhood.

According to Lévinas (2005/1961), our original identity is the basis for our existence. The finding in this study points to a culture of efficiency and rationality in the care that seems to focus more on specific tasks and that the care was performed in a pragmatic way. This may minimizes opportunities for the caregiver to truly encounter the patient and to meet their sublime wish, which could give the possibility for the patient to feel valued as a human being and to be someone, an “I can.” During the encounter with the caregiver, the patient cannot choose whether or when the encounter will lead to confirmation; it was found to be unpredictable. The confirmation may occur in the next encounter with the caregiver, but it could be absent as well. Patients may not know when the caregiver will appear, or if the caregiver will invite them into an intersubjective dialogue. The caregivers can be somewhere near without truly paying attention to and acknowledging the patient. This means that the patient may still be feeling lonely, even if the caregiver is present. Dahlberg (2007) describes how one can feel lonely, even if there are other people around. Being involuntarily lonely and not belonging to anyone are ways of “not being”. This can be compared to feelings of standing outside the human community, as found in this study.

The caring relationship is important to avoid unnecessary suffering. Human contact and feeling connected with the caregiver are important. In this study, suffering was experienced when the caregiver was absent in the care of the patient. A study by Custers, Westerhof, Kuin, and Riksen-Waraven (2010) showed that residents in nursing homes needed fulfilment in caring relationships and that the caring relationships contributed to the residents’ well-being. Such caring relationships were built upon dialogue between the patient and the caregiver. Caring relationships also have been identified as important in the care of patients suffering from dementia. A study by Rundqvist and Severinsson (1999) showed three factors that are important for a caring relationship: touch, mutual confirmation, and the caregiver’s values in the caring culture. The way that caregivers communicate in the encounter may therefore be essential to avoid unnecessary suffering.

Building a dialogue based on encounters between the patients and the caregivers is probably essential to avoiding experiences of insecurity, loneliness, and alienation, but these mutual encounters built upon dialogue seemed to be rare in this study. The lack of mutuality will probably obstruct the confirmation of the patient as an individual, which may be required to maintain a human identity. The care organization and the caregiver did not give the patient enough space and time that were needed to be a human among humans, that is, a sense of belonging in a human community (Bengtsson, 1998). All of this led to inadequate care. Malmedal, Ingebrigtsen, and Saveman (2009) showed in a Norwegian study of 16 nursing homes a high extent of different types of inadequate care. The high extent confirmed that the result was not isolated uncaring acts, but rather was a common part of life in these nursing homes. Although the most common uncaring acts were of an emotional and negligent character, such as talking disrespectfully or ignoring the patients, in some ways the patients were exposed to unnecessary suffering.

### Conclusion and clinical implications

This study reveals that persons with dementia with a manifest care need who live at home and people who are cared for in a nursing home experience suffering. Suffering from care increased the elderly patients’ feelings of insecurity, loneliness, and alienation; this seemed to be the foundation for patients’ experiences of being outside of a human community.

The suffering sometimes was caused by various caring actions, that is, it was unnecessary suffering. The suffering caused by care that arose was due to caregivers’ inability to be present, to show their face, and to truly meet the patient.

There was a lack of knowledge and understanding about the patient’s lifeworld. The care organization seemed deficient and did not rest on patients’ perspectives; that is, it was not based on a holistic approach where a patient’s lifeworld is taken into account. To avoid unnecessary suffering, it is important that there is a focus on caring directed to the actual meeting between the patient and the caregiver, as well as on what this means for the patient’s experience of well-being in terms of security, community, and belonging. To accomplish this, some important actions probably are required, such as nursing guidance for both staff and managers, to highlight the importance of each encounter with the patient. By working with a moral care organization that can promise dialogue between patients and caregivers, patients probably will be supported in finding their own rhythm of life, which includes a sense of belonging somewhere with someone. This would certainly help them to carry out various life projects, which in this context, for example, could mean inviting someone for a talk and a cup of coffee or being invited for a walk with someone that may include an exchange of feelings and thoughts. The organization, in this way, would support a healthy process in which the patient’s sense of well-being can be promoted.

## References

[CIT0001] Almberg B, Grafström M, Winblad B (1997). Caring for a demented elderly person—Burden and burnout among caregiving relatives. Journal of Advanced Nursing.

[CIT0002] Andersson I, Pettersson E, Sidenwall B (2007). Daily life after moving into a care home—Experiences from older people, relatives and contact persons. Journal of Clinical Nursing.

[CIT0003] Bengtsson J (1998). *Phenomenological excursions: The human being and science from a lifeworld perspective* [Fenomenologiska utflykter: människa och vetenskap ur ett livsvärldsperspektiv].

[CIT0004] Bergland Å, Kirkevold M (2001). Thriving—A useful theoretical perspective to capture the experience of well-being among frail elderly in nursing homes. Journal of Advanced Nursing.

[CIT0005] Berglund A. L (2007). Satisfaction with caring and living conditions in nursing homes: Views of elderly persons, next of kin and staff members. International Journal of Nursing Practice.

[CIT0006] Berglund M, Westin L, Svanström R, Johansson Sundler A (2012). Suffering caused by care—patients’ experiences in hospital settings. International Journal of Qualitative Studies on Health and Well-being.

[CIT0007] Brown C. J, Shlosberg E (2006). Attachment theory and dementia: A review of the literature. Aging & Mental Health.

[CIT0008] Cook G (2006). The risk to enduring relationships following the move to a care home. International Journal of Older People Nursing.

[CIT0009] Custers A. F, Westerhof G. J, Kuin Y, Riksen-Waraven M (2010). Need fulfillment in caring relationships: Its relation with wellbeing of residents in somatic nursing homes. Aging & Mental Health.

[CIT0010] Dahlberg K (2007). The enigmatic phenomenon of loneliness. International Journal of Qualitative Studies on Health and Well-being.

[CIT0011] Dahlberg K, Dahlberg H, Nyström M (2008). Reflective lifeworld research.

[CIT0012] Dahlberg K, Segesten K (2010). *Health and caring in theory and practice* [Hälsa och vårdande i teori och praxis].

[CIT0013] Eriksson K (1994). *The suffering human being* [Den lidande människan].

[CIT0014] Eriksson K (1997). Understanding the world of the patient, the suffering human being: The new clinical paradigm from nursing to caring. Advanced Practice Nurse Quarterly.

[CIT0015] Eriksson K (2002). Caring science in a new key. Nursing Science Quarterly.

[CIT0016] Fagerström L, Eriksson K, Bergbom-Engberg I (1998). The patients perceived caring needs as a message of suffering. Journal of Advanced Nursing.

[CIT0017] Gijsberts M. J, van Der Steen J. T, Muller M. T, Hertogh C. M, Deliense L (2013). Spiritual end of life care in Dutch nursing homes: An ethnographic study. Journal of the American Medical Directors Association.

[CIT0018] Giorgi A (2009). The descriptive phenomenological method in psychology: A modified Husserlian approach.

[CIT0019] Heaton J (2004). Reworking qualitative data.

[CIT0020] Hellström I, Nolan M, Lundh U (2007). Sustaining “couplehood”: Spouses’ strategies for living positively with dementia. Dementia.

[CIT0021] Hjaltadóttir I, Gustafsdóttir M (2007). Quality of life in nursing homes: Perceptions of physically frail elderly residents. Scandinavian Journal of Caring Sciences.

[CIT0022] Jansson W, Nordberg G, Grafström M (2001). Patterns of elderly spousal caregiving in dementia care: An observational study. Journal of Advanced Nursing.

[CIT0023] Kasén A, Nordman T, Lindholm T, Eriksson K (2008). When the patient suffers from care—carers portrayal of patients’ care suffering [Då patienten lider av vården—vårdares gestaltning av patientens vårdlidande]. Vård i Norden.

[CIT0024] Kitwood T (1997). The experience of dementia. Aging & Mental Health.

[CIT0025] Lévinas E (2005). Totality and infinity: An essay on exteriority.

[CIT0026] Malmedal W, Ingebrigtsen O, Saveman B. I (2009). Inadequate care in Norwegian nursing homes—as reported by nursing staff. Scandinavian Journal of Nursing Sciences.

[CIT0027] Merleau-Ponty M (2002). Phenomenology of perception.

[CIT0028] Nordberg G, Wimo A, Jönsson L, Kåreholt I, Sjölund B. M, Lagergren M, von Strauss E (2007). Time use and costs of institutionalised elderly persons with or without dementia: Results from the Nordanstig cohort in the Kungsholmen Project—a population based study in Sweden. International Journal of Geriatric Psychiatry.

[CIT0029] Nygård L, Borell L (1998). A life-world altering meaning: Expressions of the illness experience of dementia in everyday life over 3 years. The Occupational Therapy Journal of Research.

[CIT0030] Nygård L, Johansson M (2001). The experience and management of temporality in five cases of dementia. Scandinavian Journal of Occupational Therapy.

[CIT0031] Nygård L, Starkhammar S (2003). Telephone use among non-institutionalized persons with dementia living alone: Mapping out difficulties and response strategies. Scandinavian Journal of Caring Science.

[CIT0032] Oosterveld-Vlug M. G, Pasman H. R, van Gennip I. E, Muller M. T, Willems D. L, Onwuteaka-Philipsen B. D (2013). Dignity and the factors that influence it according to nursing home residents: A quality interview study. Journal of Advanced Nursing.

[CIT0033] O’Shaughnessy M. O, Lee K, Lintern T (2010). Changes in the couple relationship in dementia care: Spouse carers’ experiences. Dementia.

[CIT0034] Phinney A, Chesla C. A (2003). The lived body in dementia. Journal of Aging Studies.

[CIT0035] Rundqvist E. M, Severinsson E. I (1999). Caring relationships with patient suffering from dementia—an interview study. Journal of Advanced Nursing.

[CIT0036] SBU (2008). Dementia—etiology and epidemiology, a systematic review.

[CIT0037] Söderlund M (2004). *As hit by a hurricane: Relatives’ lives when a loved one has dementia* [Som drabbad av en orkan, anhörigas tillvaro när en närstående drabbas av demens].

[CIT0038] Svanström R, Dahlberg K (2004). Living with dementia yields a heteronomous and lost existence. Western Journal of Nursing Research.

[CIT0039] Svanström R, Sundler A. J (2013). Gradually losing one’s foothold—a fragmented existence when living alone with dementia. Dementia.

[CIT0040] Wadensten B (2007). Life situation and daily life in a nursing home as described by nursing home residents in Sweden. International Journal of Older People Nursing.

[CIT0041] Westin L, Danielson E (2007). Encounters in Swedish nursing homes: A hermeneutic study about residents’ experiences. Journal of Advanced Nursing.

